# Measles, rubella, mumps and chicken pox seroprevalence of health workers in a second level hospital

**DOI:** 10.1093/eurpub/ckac131.397

**Published:** 2022-10-25

**Authors:** I Guerrero Fernández de Alba, E Soler Iborte, R Padilla Matas, J Diaz Campanón, M Rivera Izquierdo, J Perez de Rojas

**Affiliations:** Preventive Medicine and Public Health, Hospital Universitario Clínico San Cecilio, Granada, Spain

## Abstract

Healthcare workers are a professional group subject to a risk of occupational exposure to a variety of infectious agents. From a public health perspective, their immune status has a great impact on the worker’s own health, on the patients and on the general population. Measles, mumps, rubella, and chickenpox are vaccine-preventable diseases caused by viruses. Seroprevalence surveys are a powerful evaluation tool that provide information on the frequency, distribution, and dynamics of communicable diseases. In this study, the prevalence of immunity to measles, mumps, rubella, and varicella viruses was analyzed in healthcare workers in a General Hospital of Granada (Spain). A cross-sectional study examining the seroprevalence was carried out in a population of health professionals incorporated into the General Hospital between January 2021 and February 2022. 260 professionals were studied, classified into groups according to age: 20-29, 30-39. Serum determination of IgG to measles virus was performed using a marketed chemiluminescent immunoassay. The resulting seroconversion rates were: 66.54% measles, 89.75% rubella, 84.62% mumps, and 88.08% chickenpox. The lowest rates were observed for measles, resulting in a minimum among professionals between 20-29 years of age, with a seroconversion rate of 65.4%. In general, women had a higher percentage of antibodies against measles. The highest rates were for the varicella virus, reaching up to 93.18% among health professionals between 30-39 years old. Considerable decrease in titers of antibodies against measles is observed in healthcare workers, especially in the age group 20-29 years, which may be due to the loss of serological protection as time goes by since vaccination with the 2nd dose of Triple Viral, possibly due to the absence of contact with the wild virus. It will be necessary to assess the need for new vaccination strategies in certain population groups such as healthcare workers based on their risk of exposure.

**Key messages:**

• It will be necessary to assess the need for new vaccination strategies in certain population groups.

• More seroprevalence studies are necessary to update the status of protection against infectious disease.

